# Prediction of protein structural class with Rough Sets

**DOI:** 10.1186/1471-2105-7-20

**Published:** 2006-01-14

**Authors:** Youfang Cao, Shi Liu, Lida Zhang, Jie Qin, Jiang Wang, Kexuan Tang

**Affiliations:** 1 Plant Biotechnology Research Center, Fudan-SJTU-Nottingham Plant Biotechnology R&D Center, School of Agriculture and Biology, Institute of Systems Biology, Shanghai Jiao Tong University, Shanghai 200030, China; 2 State Key Laboratory of Genetic Engineering, School of Life Sciences, Fudan-SJTU-Nottingham Plant Biotechnology R&D Center, Morgan-Tan International Center for Life Sciences, Fudan University, Shanghai 200433, China

## Abstract

**Background:**

A new method for the prediction of protein structural classes is constructed based on Rough Sets algorithm, which is a rule-based data mining method. Amino acid compositions and 8 physicochemical properties data are used as conditional attributes for the construction of decision system. After reducing the decision system, decision rules are generated, which can be used to classify new objects.

**Results:**

In this study, self-consistency and jackknife tests on the datasets constructed by G.P. Zhou (Journal of Protein Chemistry, 1998, 17: 729–738) are used to verify the performance of this method, and are compared with some of prior works. The results showed that the rough sets approach is very promising and may play a complementary role to the existing powerful approaches, such as the component-coupled, neural network, SVM, and LogitBoost approaches.

**Conclusion:**

The results with high success rates indicate that the rough sets approach as proposed in this paper might hold a high potential to become a useful tool in bioinformatics.

## Background

Because there is a gap between sequence and structure, the prediction of protein structural classes is still a hot research field today. One protein usually can be classified into one of the four structural classes: all-α, all-β, α/β and α+β. Many different algorithms and efforts have been made to address this problem so far. A review about prediction of protein structural class and subcellular locations by Chou [1] presented this problem systematically, and introduced and compared some existing methods.

In 1986, Klein and Delisi [2] first put forward the prediction of protein structural classes, and shortly afterward, Klein [3] brought discriminate analysis method to this problem. A new weighting method [4] was proposed to predict protein structural classes from amino acid composition in 1992. After that, another new method, called maximum component coefficient method, was proposed by Zhang and Chou [5], which had a higher correct rate than other methods. Later, a new neural networks based algorithm [6] was developed that considers six hydrophobic amino acid patterns together with amino acid compositions, and a cross-validation test was used to verify the accuracy of this method. Chou [7] brought a novel approach to predict protein structural class in a (20-1)-D amino acid composition space, which takes into account the coupling effect among different amino acid components of a protein by a covariance matrix. A method based on the scale of Mahalanobis distance is proposed by Chou and Zhang [8] in 1994, and it also incorporates the correlative effect among different amino acids automatically. Chou *et al*. [9] proposed the component-coupling algorithm that took into account the coupling effect among different amino acid components. This method was ever thought to be one of the most accurate algorithms to predict protein structural classes. Later, Zhou and Assa-Munt [10] revealed the subtle relation among the Mahalanobis algorithm, the component-coupled algorithm, and the Bayes decision rule, and that the component-coupled algorithm is much more efficient than the simple geometry algorithm in protein structural class prediction.

In 2001, Cai *et al*. [11] introduced Support Vector Machine, a machine learning method based on statistical learning theory, to deal with this problem. Functional domain composition was introduced by Chou and Cai [12] to predict protein structural class, meanwhile they introduced three other classes: μ (multi-domain), σ (small protein), and ρ (peptide), so that the prediction was expanded from 4 to 7 classes and that sequence-related and function-related features could both be incorporated into the predictor. Recently, Shen *et al. *[13] introduced supervised fuzzy clustering approach to this problem. Another recently developed powerful method, LogitBoost, was proposed by Fend *et al. *[14], which classifies protein through combining many weak classifiers together in order to build a stronger classifier; and actually LogitBoost has performed very well.

In this study, we developed a method based on supervised learning approach, Rough Sets [15], to predict protein structural classes. Two datasets constructed by Zhou [16] from SCOP were used to verify and test the efficiency of Rough Sets. The Rough Sets platform we used was the Rosetta system developed by Øhrn [17], which was a publicly available platform for data mining with Rough Sets. Amino acid composition and 8 values of physicochemical properties were extracted from primary sequences of datasets to construct the decision table.

## Results

In order to verify the performance of this rough sets based method, we carried out self-consistency test and cross-validation based on jackknife test to evaluate the prediction results. Both are thought to be the most rigorous and objective methods for evaluation of prediction.

Self-consistency tests are performed against the two datasets, and the results of self-consistency tests are showed in Table 2. All the percentages of correct prediction on both datasets reach 100%, which is the same as the results of SVM method [11]. The results indicated that Rough Sets captured the characteristics between sequences and their classes through amino acid composition and physicochemical properties.

Jackknife test is performed on the datasets. The results are illustrated in Table 3.

Through the reduction of decision tables, two sets of decision rules are generated as classifiers. The classifier trained by 277 domains contains 46651 decision rules in total, and the one of 498 domains contains 52474 decision rules. The distribution of 4 structural classes in decision rules is shown in Table 4. These rules can be used to classify new protein sequences to the 4 structural classes.

## Discussion

**Figure 1:**
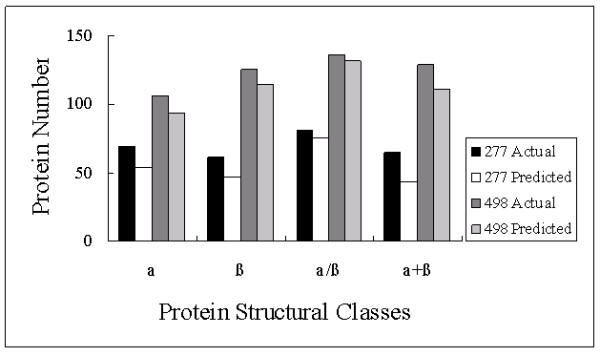
The comparisons between actual objects number and predicted number of 4 protein structural classes.

From the results of jackknife tests, we can see that α/β class has the highest accuracy, no matter compared with whichever class or algorithms. This may be related to the proportion of α/β class in the training sets in which α/β class occupied the biggest part, as shown in Figure 1. As a supervised learning method, it makes it easier to capture characteristics that feed more training objects to Rough Sets.

Although the average accuracy of 498 domains of Rough Sets is slightly lower than the SVM, they are still much better than others. So, from the results of jackknife, we can conclude that the performance of Rough Sets should have exceeded the component-coupled algorithm and neural networks in this study, and parallel with SVM algorithm. In addition, since the extraction of data, coupling effects among amino acids are not considered yet, we only take into account the amino acid composition and 8 physicochemical properties which may influence the secondary structure of proteins. Based on this point, we consider it is reasonable to believe that the algorithm based on Rough Sets still has potential to improve.

Rough Sets is a very promising method in bioinformatics. However, a quick search of biological literatures shows that Rough Sets are still seldom used in bioinformatics, except for some applications in medical and health related fields. One obstacle for the application of Rough Sets in bioinformatics may be the large amounts of biological data and the comparatively slow computational speed of Rough Sets algorithm. The computation of Discernibility has a time complexity of *O*(*n*^*2*^), which is still higher than many other algorithms in bioinformatics.

There are several factors that may affect the precision of prediction based on Rough Sets. One of them is the conditional attributes, and another is the scale of datasets. The selection of conditional attributes must reflect the relation between sequences and their structural classes. If a set of conditional attributes does not make this bridge, Rough Sets can not induce effective rules from the decision system. From this study, we can see that amino acid compositions and physicochemical properties certainly can be used to discriminate protein sequences from different structural classes. However, the conformation of secondary structure of protein is very complex, and there are still other factors that may influence this process and that can be taken into account to improve this method.

In theory, the more objects in the dataset, the more accurate the prediction would be; in other words, the more information there is about the problem, the more likely to induce useful rules from it. We have seen that the accuracy of 498 domains is much higher than the 277 domains. If possible, we can get more accurately predicted results through constructing as large a dataset as possible to train a Rough Sets model with a proper attribute set. In the future study, we will deal with larger datasets and improve the performance of Rough Sets based method.

## Conclusion

In summary, we reported a successful method based on Rough Sets theory to predict protein structural classes. We used two datasets constructed by Zhou [16] to test this new method. High prediction accuracies have been achieved through self-consistency and cross-validation test. This suggests that the rough sets approach holds a high potential to become a useful tool in bioinformatics. Furthermore, as elucidated in a recent review by Chou [18], the past progresses in protein structural class prediction had a series of impacts to the development for predicting many other attributes of proteins. We believe that the current rough sets approach might also stimulate the development in predicting other protein attributes, such as subcellular location [19-21], membrane protein type [22-24], and enzyme family classification [25], among many others.

## Methods

### Datasets

Two datasets of protein domain sequences used in this study came from Zhou [16], which were extracted respectively from SCOP database [26], one consisted of 277 sequences, and another 498 sequences. The composition of 4 domain classes is listed in the Table 1 respectively.

We used the two datasets to test our method through self-consistency test, jackknife test, and induced decision rules from datasets, and compared the total accuracy and accuracies of each structural class with other algorithms.

### Rough Sets

Rough sets theory is a machine learning method, which is introduced by Pawlak [15] in the early 1980s as a tool of representing and reasoning about imprecise and uncertain data. Rough sets theory distinguishes between objects based on the concept of indiscernibility, and deals with the approximation of sets by means of binary relations that is typically constructed from empirical data. It constitutes a mathematical framework for inducing minimal decision rules from training examples. Each 'if-then' rule induced from decision table identifies a minimal set of features discriminating one particular example from other classes. The set of rules induced from all training examples constitutes a classificatory model capable of classifying new objects. A typical application of rough sets method usually includes three steps: construction of decision tables, model induction, and model evaluation respectively. The detailed description of each step in the method is given in the next three sections.

### Construction of decision system

The 3D structure of proteins is uniquely determined by their amino acid sequences, i.e. the primary sequences. In order to predict the structural classes of proteins, primary sequences have been converted into numerical vectors, in other words, features information has been extracted from primary sequences as the representation of proteins. There are many ways to extract features from amino acid sequences for representing the essential characteristics of proteins. In this paper, we used amino acid compositions and physico-chemical properties as the features to describe amino acid sequences, i.e. conditional attributes set.

Because of the degenerate nature of the sequence-structure relationship, the number of their folding patterns is quite limited, despite that the number of protein sequences is extremely large. In fact, according to their chain folding patterns, proteins are usually folded into one of the following four structural classes: all-α, all-β, α/β, and α+β. In this paper, we build attribute set using amino acid composition and physicochemical properties that are computed with the method of SAPS [27], a program from ExPASy web site.

Given a decision system A = (*U*, *A*∪{*d*}), here *A *is called the *conditional attributes *set, and *d *∉ *A *is called *decision attribute*. The elements of the universe *U *are called objects. Each object in *U *has an according decision value with it in {*d*}. In this research, conditional attributes set *A *is made up of two parts, the first part is *S*_*1 *_= {A, R, N, D, C, Q, E, G, H, I, L, K, M, F, P, S, T, W, Y, V}, which is composed of compositional percentages of the 20 amino acids in primary sequences, and the second part is *S*_*2 *_= {KR, ED, AGP, KRED, KR-ED, FIKMNY, LVIFM, ST}, the 8 physicochemical properties: positive charge (KR), negative charge (ED), total charge (KRED), net charge (KR-ED), major hydrophobic (LVIFM), and groupings ST, AGP, and FIKMNY. So we can write *A *as: *A *= *S*_*1 *_∪ *S*_*2*_, and the *decision attribute *{*d*} = { all-α, all-β, α/β, α+β}.

### Model induction

Rough sets based models are built on the concept of indiscernibility. Given a decision system A = (*U*, *A*∪{*d*}), we define *IND*_A_(*A*,*x*,*d*) to be the set of objects that are indiscernible from *x *with respect to the decision attribute *d*. From the definition of indiscernibility we derive for each object *x *∊ *U *the set of reducts *RED*_A_(*x*,*d*) to be the set of minimal sets of attributes *B *⊆ *A *such that *IND*_A _(*B*,*x*,*d*) = *IND*_A _(*A*,*x*,*d*). Hence, a reduct of *x *is a minimal set of attributes *B *with the same discriminatory power as *A*. Finding the set of all reducts is a NP-hard problem, however, there are heuristics that compute a sufficient number of reducts in an acceptable computing time. Since real-world data almost always is polluted with noise, methods finding approximate reducts that reveal the underlying, general pattern in the data have also been developed.

### Model evaluation and application

In a self-consistency test, training sets will be predicted with those decision rules trained by them. The accuracy of self-consistency test can tell us how well the rules captured the characteristics of training sets.

As elucidated in a comprehensive review [28], jackknife test is deemed to be the most objective and rigorous way to estimate the performance of a classifier. Each object in the dataset will in turn be the test set, and the left as training set. In other words, each protein of the dataset will be predicted once by the classifier trained with the left proteins. After this process, we can average all iterations to obtain an unbiased number of performance estimates.

In this study, self-consistency tests and jackknife tests are performed against two datasets, and the results are compared with other algorithms, including component coupled algorithm [16], neural networks [29] and SVM [11].

### Algorithm and implementation

Except for several Perl scripts we wrote to deal with protein sequences and extract features from them, all of computation concerning on Rough Sets is implemented on Rosetta system, which is a toolkit for data mining and knowledge discovery using rough sets. Rosetta system is implemented with C++, and is ported to a variety of platforms. We used their Win32 copy, which has a GUI front end developed with the MFC (Microsoft Foundation Classes). More detailed descriptions and cases of application on Rosetta system can be found in Øhrn's PhD dissertation [17].

**Figure 2:**
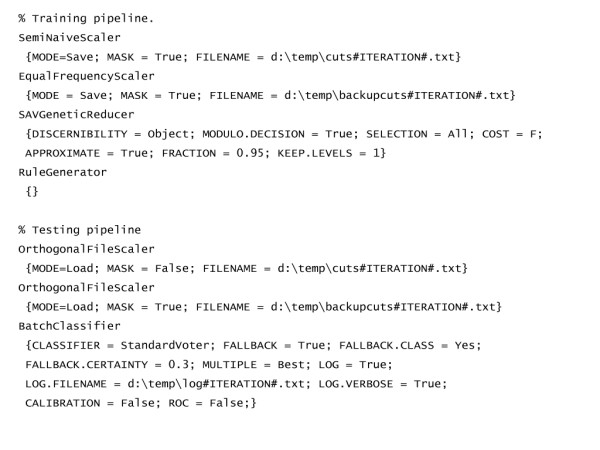
Pipeline script for cross-validation test on Rosetta system.

Rosetta system includes a large variety of typical algorithms that can be used in almost each step of data mining with rough sets. Users can choose appropriate algorithms for various situations. Another advantage of Rosetta system is that users can perform very complicated cross-validation tests based on a user defined script. In this study, we write a pipeline script for performing CV test on Rosetta (Figure 2). With this script, we perform jackknife test of cross-validation over the datasets described above. For our datasets, two critical steps that may affect predicting results are discretization and reduction. We use Semi-Naive algorithm (SemiNaiveScaler) and genetic algorithm (SAVGeneticReducer) respectively for the two steps.

As discussed above, computing all reducts of a decision table is a NP-hard problem. Many alternative algorithms have been developed for computing of approximate reducts.

## Authors' contributions

YC proposed the idea of using rough sets to perform protein structural class prediction, and wrote most of script programs. SL and JW helped to refine the idea. LZ carried out data statistics and verification. JQ found out and prepared the datasets for computation used in this study. KT is responsible for guiding the whole project. All authors have read and approved this final manuscript.

## Figures and Tables

**Table 1:** The composition of two datasets in this study

Dataset	All-α	All-β	α/β	α+β	Total
277 domains	70	61	81	65	277
498 domains	107	126	136	129	498

**Table 2:** Results of self-consistency test

Dataset	Algorithm	Rate of correct prediction for each class				Overall rate of accuracy
			
		All-α	All-β	α/β	α+β	
277 domains	Component coupled	95.7%	93.4%	95.1%	92.3%	94.2%
	Neural network	98.6%	93.4%	96.3%	84.6%	93.5%
	SVM	100%	100%	100%	100%	100%
	Rough Sets	100%	100%	100%	100%	100%

498 domains	Component coupled	95.8%	95.2%	94.9%	95.4%	95.8%
	Neural network	100%	98.4%	96.3%	84.5%	94.6%
	SVM	100%	100%	100%	100%	100%
	Rough Sets	100%	100%	100%	100%	100%

**Table 3:** Results of jackknife test

Dataset	Algorithm	Rate of correct prediction for each class				Overall rate of accuracy
			
		All-α	All-β	α/β	α+β	
277 domains	Component coupled	84.3%	82.0%	81.5%	67.7%	79.1%
	Neural network	68.6%	85.2%	86.4%	56.9%	74.7%
	SVM	74.3%	82.0%	87.7%	72.3%	79.4%
	Rough Sets	77.1%	77.0%	93.8%	66.2%	79.4%

498 domains	Component coupled	93.5%	88.9%	90.4%	84.5%	89.2%
	Neural network	86.0%	96.0%	88.2%	86.0%	89.2%
	SVM	88.8%	95.2%	96.3%	91.5%	93.2%
	Rough Sets	87.9%	91.3%	97.1%	86.0%	90.8%

**Table 4:** Statistics of decision rules

	277 domains	Percentage	498 domains	Percentage
α	11711	25.10%	12744	24.29%
β	10250	21.97%	11211	21.36%
α/β	11886	25.48%	13771	26.34%
α+β	12804	27.45%	14748	28.11%

Total	46651		52474	
